# Systemic Lupus Erythematosus in the Elderly That Debuts With an Organic Manifestation of Lupus Nephritis

**DOI:** 10.7759/cureus.28746

**Published:** 2022-09-03

**Authors:** Anosh Khan, Tirtha Sawant, Zahra Deen, Wasay Humayun, Youshay Humayun

**Affiliations:** 1 Nephrology, Spartan Health Sciences University School of Medicine, Vieux Fort, LCA; 2 Nephrology, Elmhurst Hospital, Elmhurst, USA; 3 Nephrology, Swedish Hospital Part of NorthShore University Healthsystem, Chicago, USA

**Keywords:** hispanic, case report, lupus nephritis, prednisone, mycophenolate mofetil, late-onset sle

## Abstract

Systemic lupus erythematosus (SLE) is a systemic autoimmune condition with many clinical presentations. It is classically seen in young to middle-aged females and can present with cutaneous, renal, serosal, hematological, joint, and/or neurological manifestations at the time of diagnosis or may develop over the course of the disease. Late-onset SLE or SLE in the elderly is a subtype that differs from the classic SLE in age group, clinical presentation, involvement of organs, and severity. Here, we present the case of a geriatric Hispanic male noted to have worsening renal function. The patient was diagnosed with lupus nephritis (LN) upon obtaining serological markers and renal biopsy. LN, a renal sequela of SLE, presents with a full-house immunofluorescence pattern. LN, along with high titers of the antinuclear antibody (ANA) and/or anti-double-stranded DNA (anti-dsDNA) antibody, is an effective tool to diagnose SLE in patients without extrarenal manifestations of the disease. The patient was managed with glucocorticoids and mycophenolate mofetil therapy, which led to a rapid downtrend of creatinine, resulting in stabilization of renal function and deferring the need for a hemodialysis. This case highlights the topic of late-onset SLE presenting with LN in geriatric patients.

## Introduction

Systemic lupus erythematosus (SLE) is a multi-systemic, autoimmune disease with unknown etiology, varied clinical and laboratory findings, and a wide variable course and prognosis [1,2]. It may present as a single episode or show a relapsing and remitting course that may be associated with or may be independent of lupus flares [3]. SLE is classically seen in reproductive-aged females, and its generalized symptoms include fever, arthralgia, weight loss, and fatigue. In addition, the majority of patients report dermatological changes like malar rash, alopecia, and oral ulcers. Cardiopulmonary manifestations include dyspnea, pulmonary hypertension, pleuritis, pericarditis, and myocarditis. SLE also commonly presents with anemia, thrombocytopenia, and/or leukopenia. Lastly, patients with SLE frequently experience but are not limited to headaches, anxiety, depression, psychosis, and seizures.

Late-onset SLE represents a specific subgroup of SLE, and although there is no strict age cutoff, 50 years is commonly used as the minimum age of disease onset [4,5]. According to several epidemiological reports, late-onset SLE has been noted to be present in patients over the age of 50-60 in the past 25 years. Although it is rare and infrequent, the term late-onset SLE or SLE in the elderly has been used to differentiate it from the classic SLE. However, in the case of male geriatric patients, the diagnosis is often overlooked due to the low incidence and atypical manifestations of the disease. Fewer than 10% of patients may first present with a single, generalized (often severe) manifestation from one organ system, such as the kidneys or central nervous system (organ-dominant disease) [6]. Out of all the solid organs involved, renal involvement in SLE is one of the most common and feared manifestations of this disease as it is related to high morbidity and increased mortality rate, especially in lupus nephritis (LN), which is a highly specific finding [7]. LN is characterized by a full house immunofluorescence pattern, i.e., positivity for IgG, IgA, IgM, C3, and C1Q on the kidney biopsy. Although LN can develop at various time points during the disease course of SLE, it usually develops within five years of SLE onset [8,9] and, more commonly, at the onset of SLE (in approximately 50% of cases) [10]. Therefore, we report the case of a geriatric male in whom late-onset SLE with LN was discovered as an etiology of worsening renal function, despite the patient experiencing no extrarenal manifestations of the disease over the course of his life. We hope that this report serves as an aid to future physicians during the diagnosis and management of this condition.

## Case presentation

**Figure 1:**
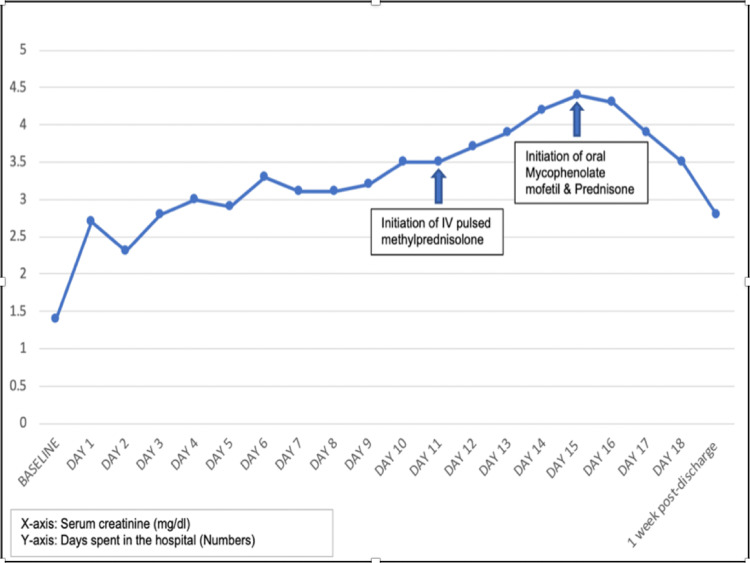
Serum creatinine trend.

A 69-year-old Hispanic male with a past medical history of hypertension, hyperlipidemia, Hashimoto’s thyroiditis, benign prostatic hyperplasia, autoimmune hepatitis, and chronic kidney disease stage 3A presented to the ED with a fever of 103 F, chills, and non-productive cough for the past three days. Medications before admission were amlodipine 10 mg, atorvastatin 10 mg, finasteride 5 mg, hydrochlorothiazide 25 mg, levothyroxine 75 mcg, and aspirin 81 mg. On the day of admission, the patient’s physical examination was unremarkable except for bilateral pedal edema. His blood pressure was 135/81 mmHg with a pulse of 66 beats per minute (bpm). Laboratory examination revealed mild leukocytosis (11.98 × 109/L), mild lactic acidosis (2.4 mmol/L), elevated inflammatory marker (C-reactive protein [CRP], 76.8 mg/L, procalcitonin, 3.03 ng/ml), and elevated creatinine (2.7 mg/dl from a baseline of 1.4 mg/dl). The patient’s chest X-ray showed no acute cardiopulmonary abnormality. Although it did not show any consolidation, he was empirically started on ceftriaxone 1 g and azithromycin 250 mg for the next 24 hours due to clinical suspicion of community-acquired pneumonia (CAP). An unenhanced CT scan of the chest was also ordered, which showed mediastinal lymphadenopathy but no focal consolidation. Hence, based on the findings, CAP was ruled out, and empiric antibiotics were discontinued. The next day, the patient’s renal function worsened, and he was given fluids intravenously. Renal ultrasound with post-void bladder scan showed normal bilateral kidneys without any signs of hydronephrosis and a residual volume of fewer than 60 ml. Urinalysis showed hyaline cast, 3+ urine protein, 3+ urine blood, >200 RBCs, 20-40 WBCs, and a negative urine culture. A 24-hour urine collection revealed a urine protein/creatinine ratio of 4.31 g/g and a urine microalbumin/creatinine ratio of 1872 mg/G. Over the next few days, his creatinine rose to 4.0 mg/dl, and further workup revealed a strongly positive antinuclear antibody (ANA) screen (1:1280 titers) with mildly elevated anti-RNP (1.1 AI) but normal anti-Sm, anti-SS-A (Ro), anti-SS-B (La), anti-Scl-70, anti-Jo-1, anti-dsDNA, and C3 and C4 levels. Additionally, cryoglobulins, antineutrophil cytoplasmic antibodies (ANCA), anti-GBM antibodies, and HIV and hepatitis panels were all negative. Therefore, a kidney biopsy was pursued, and while awaiting the biopsy results, he was pulsed with IV methylprednisolone 500 mg for the next four days (Figure 1).

**Figure 2:**
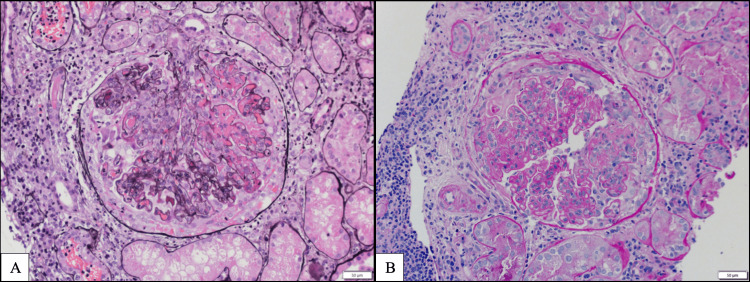
Renal biopsy: light microscopy. A: Crescentic and fibrinoid necrosis (50 μm); B: Endocapillary hypercellularity crescentic necrosis (50 μm).

The renal biopsy on light microscopy (Figure 2A-2B) was performed with tissue sections stained with H&E, periodic acid-Schiff (PAS), Masson’s trichrome, and Jones’ methenamine silver to aid morphological interpretation. Sections show renal cortex and medulla. There were 13 glomeruli; one was globally sclerotic. Nine glomeruli showed segmental-to-circumferential cellular crescents and fibrinoid necrosis with rupture of the glomerular basement membranes and fibrin extravasation into the urinary space. The remaining glomeruli appeared enlarged and showed global mesangial and endocapillary hypercellularity with intra-capillary infiltrating lymphocytes and monocytes. The glomerular basement membrane was thickened, and silver staining showed vacuolation and focal spike formation. Tubules and interstitium showed the presence of moderate diffuse interstitial inflammation composed of mainly lymphocytes and monocytes, as well as mild-to-moderate multifocal lymphocytic tubulitis. Some non-atrophic tubules exhibited acute injury with luminal ectasia and epithelial simplification. Tubular cells containing intracytoplasmic protein reabsorption droplets and many tubules showed luminal erythrocytes.

**Figure 3:**
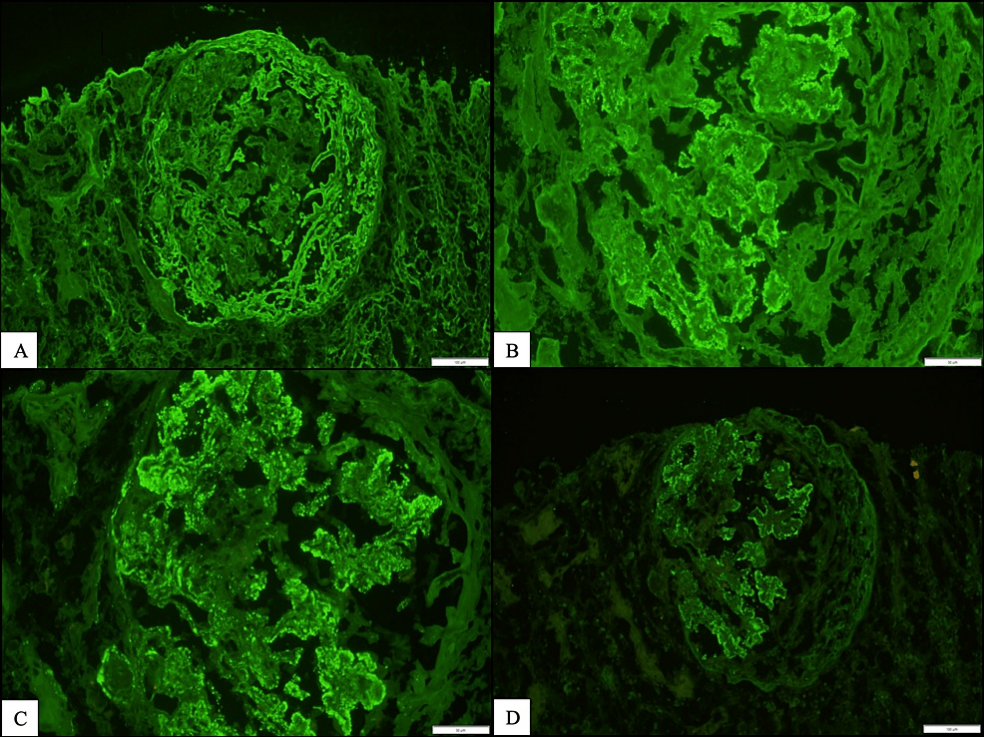
Renal biopsy: immunofluorescent microscopy. A: 3+ segmental tuft positivity for fibrinogen (100 μm); B: IgG positivity: granular global mesangial and glomerular capillary wall (50 μm); C: 2+ positivity for C1q (50 μm); and D: 3+ positivity for C3 (100 μm).

Immunofluorescent histology (Figure 3A-3D) with nine glomeruli sampled for immunofluorescence showed seven globally sclerotic. There were 2+ granular global mesangial and glomerular capillary wall positivity for IgG with a trace of IgA, 1+ IgM, 2+ C1q, 3+ C3 “full house” pattern, and negative albumin, 2+ kappa, and 2+ lambda. One glomerulus showed 3+ segmental tuft positivity for fibrinogen, and no extraglomerular positivity for IgG was seen. 

**Figure 4:**
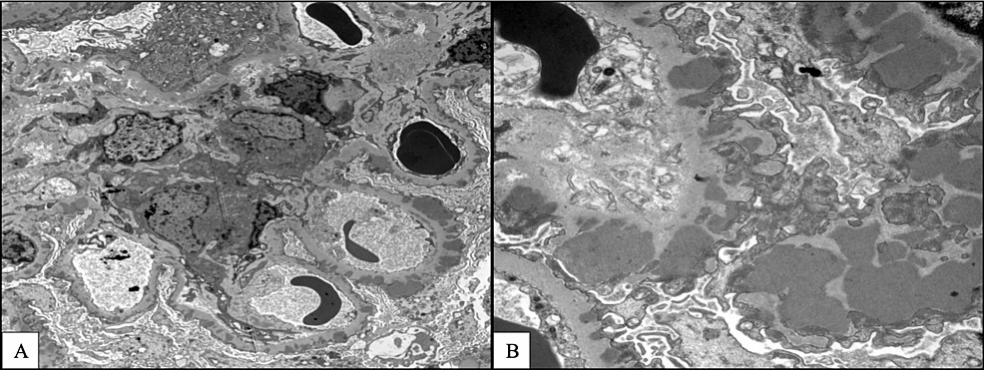
Renal biopsy: electron microscopy. A: 4 μm; and B: 1 μm demonstrating subepithelial deposits.

Electronic microscopy (Figure 4A-4B) for IgG subtypes showed 3+ granular glomerular capillary wall and mesangial staining for IgG3 with 1+ IgG1, 2+ IgG2, and 1+ IgG4, whereas immunofluorescent staining for PLA2R was negative in glomeruli.

After reviewing the biopsy results, the patient was noted to have a full house immunofluorescence pattern, i.e., positivity for all five major immunofluorescent stains, which is seen in LN. Thereafter, on the basis of the Systemic Lupus International Collaborating Clinics (SLICC) classification criteria, he was diagnosed with late-onset SLE with LN.

In line with the recommendations, the patient was started on mycophenolate mofetil 500 mg twice daily and prednisone 60 mg once daily. Following the initiation of immunosuppressive therapy, renal function stabilized, and urine output improved. On the last day of hospitalization, the patient’s creatinine improved to 3.5 mg/dl. He was discharged on mycophenolate mofetil 1500 mg twice daily and prednisone 60 mg once daily and was followed up in an outpatient setting. Lab findings from one-week post-discharge follow-up showed creatinine of 2.8 mg/dl (Figure 1).

## Discussion

SLE is a chronic multisystem disorder most commonly affecting young women [11,12]. Feldman CH et al. reported that the prevalence of SLE among females was six times higher compared to males; 38.5% of individuals with SLE were African American, 13.9% Hispanic, 4.2% Asian, 1.5% Native American, and 36.2% were white [13], highlighting the low prevalence of SLE in males and the Hispanic population. The patient in our case is a 69-year-old Hispanic male known to have late-onset SLE with biopsy-proven LN despite lacking the typical constellation of symptoms like photosensitivity, malar rash, and joint pain. In patients with an initial atypical presentation, the American College of Rheumatology (ACR) and/or SLICC classification criteria can help in diagnosis. The ACR classifications require at least four clinical and serological criteria for diagnosing SLE. However, according to SLICC classification, ANA and/or anti-double-stranded DNA (anti-dsDNA) antibody positivity in the presence of kidney biopsy consistent with LN is a criterion sufficient to diagnose SLE [14].

Late-onset SLE represents a specific subgroup of SLE, and although there is no strict age cutoff, 50 years is commonly used as the minimum age of disease onset [4,5]. This subgroup constitutes 2-12% of all patients diagnosed with SLE. Late-onset lupus differs from early-onset lupus in gender and ethnic prevalence, clinical presentation, organ involvement pattern, disease severity, and prognosis. These differences are due to age-related variation in environmental and/or host factors responsible for disease expression and to variation in sex hormones [15]. For example, clinical manifestations such as malar rash, renal disease, arthritis, and photosensitivity are less frequent in them, while serositis, cytopenias, and pulmonary involvement are more frequent [5, 16-18]. Nephritis is seen in a smaller proportion of this subgroup than in patients who present with classic SLE. For this reason, LN in late-onset SLE is considered an atypical presentation. LN presents with full-house immunofluorescence on a kidney biopsy, implying that all five major immunofluorescent stains (IgG, IgA, IgM, C3, and C1Q) on a renal biopsy are positive. In LN, it is a common observation and there is immunostaining for IgG in more than 90% of cases; IgA and IgM staining in 60-70% of cases; and C3 and C1Q in around 80% of cases [19]. The nonspecific activation of autoreactive B cells explains the polyclonal autoantibody response leading to the diagnostic hallmark of LN, the full-house pattern [20].

Aggressive management of LN is necessary because it is a significant risk factor for morbidity and mortality in SLE, and 10% of patients with LN will develop the end-stage renal disease (ESRD) [21]. The recommendations for the management of LN consisted of pulse glucocorticoids, followed by high-dose daily glucocorticoids in addition to an immunosuppressive medication [22], like cyclophosphamide, azathioprine, and mycophenolate mofetil [23,24]. Induction therapy with glucocorticoids can inhibit inflammation, whereas maintenance therapy with mycophenolate mofetil can dampen the immune-mediated damage to the kidneys. However, it is important to highlight that the clinical course of late-onset SLE is considered more benign due to less organ and systemic involvement compared to classic SLE, whose course may have a guarded prognosis due to its rapid evolution and multisystem involvement. Our patient was managed in line with the recommendations, and it is, for this reason, rapid response to immunosuppressive treatment was observed in the case, leading to a downtrend of creatinine (Figure 1).

## Conclusions

It is crucial for physicians to investigate for SLE if the renal biopsy shows a full-house immunofluorescence pattern due to the high rate of morbidity and mortality associated with LN. This should be done regardless of the patient’s age, sex, race, or absence of extrarenal manifestations, as illustrated in this case report. This underscores the importance of acknowledging any abnormal presentation of SLE when clinical suspicion remains high and conducting further investigation, including renal biopsy, if necessary. It is also vital to formulate a personalized management plan for each patient based on their comorbidities and tolerance to immunosuppressive medications. Hence, this report can help physicians accurately diagnose SLE in complex scenarios and guide in proper management with immunosuppressive therapy, leading to improvement in overall patient prognosis.
